# Correlation between membrane proteins and sizes of extracellular vesicles and particles: A potential signature for cancer diagnosis

**DOI:** 10.1002/jev2.12391

**Published:** 2023-12-05

**Authors:** Chunhui Zhai, Feng Xie, Jiaying Xu, Yuting Yang, Weiqiang Zheng, Haiyan Hu, Xianting Ding, Hui Yu

**Affiliations:** ^1^ School of Biomedical Engineering Shanghai Jiao Tong University Shanghai China; ^2^ Department of Instrument Science and Engineering School of Electronic Information and Electrical Engineering Shanghai Shanghai China; ^3^ Oncology Department Shanghai Jiao Tong University Affiliated Sixth People's Hospital Shanghai China

**Keywords:** biomarker, cancer diagnosis, extracellular vesicles and particle, liquid biopsy, membrane protein

## Abstract

Extracellular vesicles and particles (EVPs) are recognized as ideal liquid biopsy tools for cancer detection, and membrane proteins are commonly used EVP biomarkers. However, bulk analysis of EVP membrane protein biomarkers typically fails to meet the clinical requirement for diagnostic accuracy. We investigated the correlation between the membrane protein expression level, the binding kinetics to aptamers and the sizes of EVPs with interferometric plasmonic microscopy (iPM), and demonstrated the implementation of the correlative signature to determine cancer types. Using EVPs collected from both cell model and clinical plasma samples with liver, lung, breast, or prostate cancer, we found that the selective set of membrane protein expression levels of five protein markers and their binding kinetics were highly heterogeneous across various sizes of EVPs, resulting in the low overall accuracy (<50%) in cancer classification with bulk analysis of all populations. By grouping the EVPs into three subpopulations according to their sizes, the overall accuracy could be increased to about 70%. We further grouped the EVPs into subpopulations with a 10 nm interval in sizes and analysed the correlation between the membrane proteins and sizes with a machine learning algorithm. The results show that the overall accuracy to discriminate cancer types could be improved to 85%. Therefore, this work highlights the significance of size‐dependent subtyping of EVPs and suggests that the correlation between the selective set of membrane proteins and sizes of EVP can serve as a signature for clinical cancer diagnosis.

## 1 INTRODUCTION

Extracellular vesicles (EV) are a group of membrane‐enclosed phospholipid vesicles secreted by mammalian cells, including normal cells as well as cancer cells (van Niel et al., 2018). Small EVs, including exosomes and a portion of microvesicles, are a unique subset with a diameter of less than 200 nm (LeBleu & Kalluri, 2020). EVs, including small EVs, are packaged with functional molecules (i.e., proteins, amino acids and nucleic acids) of their parental cells, and play an important role in cell‐cell communication (Mathieu et al., 2019), immune response (Thery et al., 2002) and cancer metastasis (Hoshino et al., 2015; Wortzel et al., 2019). Small EVs are highly heterogeneous in membrane proteins, sizes and contents depending on the cell sources, cancer‐gene mutations and other environmental factors (Gyuris et al., 2019; Hoshino et al., 2020; Kalluri & LeBleu, 2020), making them a potential tool in cancer diagnosis (Giulietti et al., 2018; Whiteside, 2020). For example, by profiling the expression level of membrane proteins in small EVs, early diagnosis of breast cancer (Liu et al., 2019; Whiteside, 2020) and classification of cancer types (Liu et al., 2019) have been demonstrated.

With sophisticated isolation techniques, distinct size‐dependent subpopulations of exosomes, including exomere, small exosomes (Exo‐S) and large exosomes (Exo‐L) were identified, each with unique molecular biomarkers (Zhang et al., 2018). These subpopulations were collectively referred to as extracellular vesicles and particles (EVPs), and their proteins were found useful for cancer detection and determining cancer type (Hoshino et al., 2020). Conventional analytical approaches by measuring average information from the full population of EVPs would inevitably suffer from the large background noise from irrelevant subpopulations (Bordanaba‐Florit et al., 2021). Profiling the protein heterogeneity of the size‐dependent subpopulations would thus largely advance our knowledge in the mechanism of EVPs’ biological functions, as well as the development of accurate diagnostic tools (Hoshino et al., 2020). However, the major challenge to accessing the heterogeneity information is the difficulty in precisely isolating EVP subsets with specific sizes. Although there are several emerging techniques for the isolation of EVP subsets (Chen et al., 2021; Guan et al., 2019; Kowal et al., 2016; Wu et al., 2020; Zhang & Lyden, 2019; Zhang et al., 2018; Zheng et al., 2020), they typically suffer from poor efficiency, time‐consuming processes, and sophisticated sensor fabrication (Hendrix, 2021).

**FIGURE 1:**
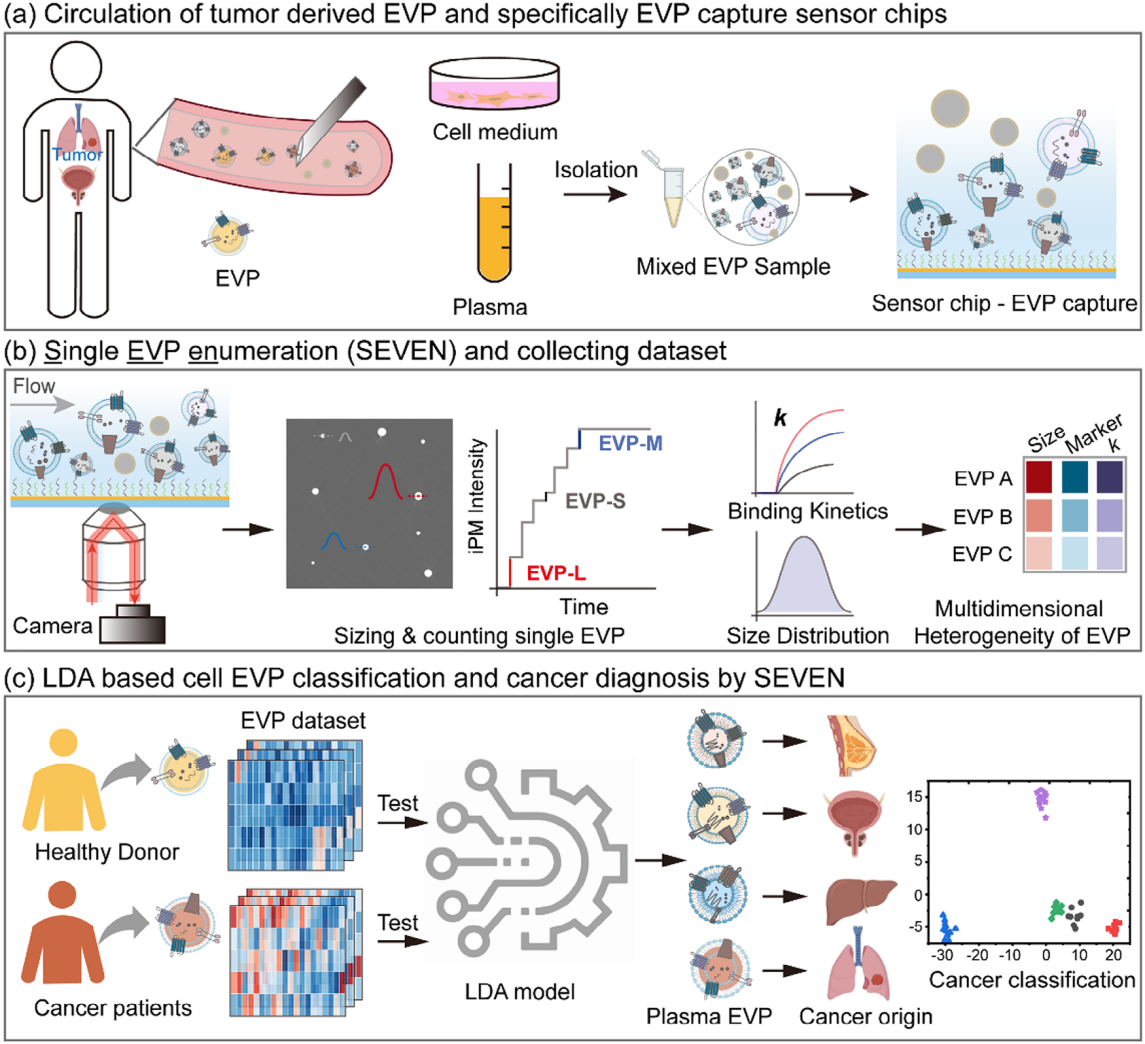
Schematics of using correlation between membrane proteins and sizes of extracellular vesicles and particles (EVPs) for cancer diagnosis. (a) The mixed EVP samples are isolated from cell culture medium or human blood samples and injected into the testing chamber. (b) EVPs binding onto specific aptamer‐coated sensors are imaged and sized with the interferometric plasmonic microscopy (iPM); image intensity and counts are quantified to construct binding curves for each subpopulation, forming a multidimensional data matrix containing the sizes, counts and binding kinetics. (c) A linear discriminant analysis (LDA) model identifies the cancer type.

Herein, we demonstrate the use of correlation between membrane proteins and sizes of EVP as a signature for cancer diagnosis. We first present an approach termed single EVP enumeration (SEVEN) to profile the protein heterogeneity in size‐dependent subsets of EVPs (Figure 1). Instead of profiling proteins on isolated size‐dependent subsets in conventional approaches, SEVEN accurately sizes single EVP captured by different aptamers (Table S1) to access the heterogeneity information. Using EVPs derived from both cancer cell lines and the plasma of cancer patients, we measured the heterogeneous expression of different membrane proteins on EVP subsets. The correlation between the selective set of membrane proteins and sizes was then analysed by a machine learning algorithm for determining cancer type.

## 2 METHODS

### 2.1 iPM system

The iPM system was built on an inverted total internal reflection fluorescence microscope (TIRFM) (Olympus IX83) using a 60 × oil immersion objective (N.A. = 1.49). The surface plasmons were stimulated via Kreschmenn configuration by a laser beam at 637 nm (OBIS 637 nm LX FP 100 mW) at a highly inclined incident angle close to the SPR dip angle. The real‐time images of small EVs were recorded by a sCMOS camera (Prime TM; Photo‐metrics). A motorized XY stage (Ludl Electronic Products, Ltd.) was incorporated on the microscope to translate the sensor chip.

### 2.2 iPM sensor chips and surface modifications

The sensor chips were 12‐542‐B (Thermo Fisher) glass coverslips coated with 3 nm of chromium and 47 nm of Au. The chips were cleaned first with deionized water for three times and then with ethanol for three times, and dried with nitrogen gas. After a quick hydrogen flame treatment, the sensor chip was immediately immersed in modification buffer for 12 h. The modification was optimized experimentally (Figure S5). The modification buffer contained 1 µM aptamer (Sangon Biotech, China) 5 µM Tris (2‐carboxyethyl) phosphine (TCEP, Sigma) and 1 µM 6‐Mercapto‐1‐Hexanol (MCH, Sigma). The chip was rinsed three times with 1× PBS buffer to remove unbound aptamers, and 50 µL of bull serum albumin (BSA, Sigma) solution (1% w/v) was added to further block the residual active sites for 5 min. For positive‐charge modification, the chip was treated with hydrogen flame and immediately submerged in a 10 mM HS‐PEG‐NH2 (10,000 Da; Nanocs) water/ethanol (1:1) solution overnight.

### 2.3 Image processing

The iPM images were processed offline with MATLAB R2018a (MathWorks). Raw images were pre‐processed by moving average with *n* = 10 frames. The differential images between two adjacent average images were reconstructed with home‐developed codes. Briefly, by calculating the radius and centre of the ring in k space, the wave vector of single EVP was determined. Deconvolution was done in k‐space using the point‐spread function obtained experimentally by aligning 30 individual images of 100‐nm silica nanoparticles to the maximum intensity point and averaging after alignment. Then the average intensity of the 3 × 3 pixels around the brightest pixel of particle images was calculated as the particle intensity.

### 2.4 Size calibration

**FIGURE 2:**
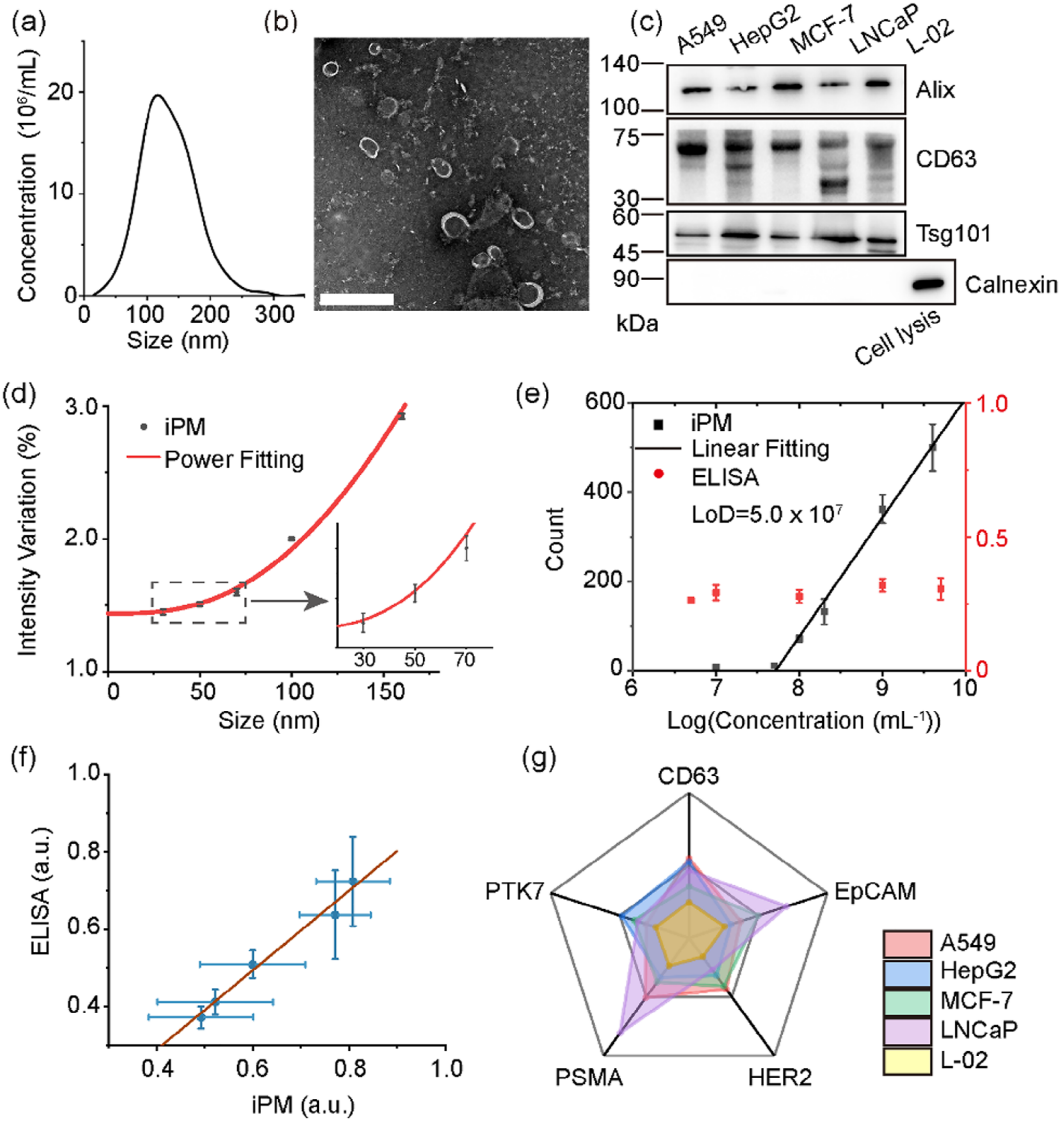
Single EVP imaging and detection. (a) Concentration and size distribution of A549‐derived EVPs measured by NTA. The EVPs samples were diluted to ∼10^7^/mL. (b) Typical TEM images of EVPs. (c) Western blot results of Alix, CD63 and Calnexin in EVPs derived from A549, HepG2, MCF‐7, LNCaP, L‐02 cell lines. (d) Theoretical (red) and experimental (black) interferometric plasmonic microscopy (iPM) intensities of silica nanoparticles of 30, 50, 70, 100 and 160 nm. (e) Sensitivity of iPM (black) and ELISA (red) for the detection of CD63‐positive EVPs from MCF‐7 cells. (f) CD63 level of EVPs from five cells lines determined by iPM and ELISA (*n* = 5). (g) EVP membrane protein level and (h) The association rates of EVPs (*n* = 3).

Silica nanoparticles (MikroNano Partikel GmbH) with the sizes of 30, 50, 70, 100 and 160 nm were used to build the size‐calibration curve. Raw silica nanoparticles were diluted with 1× PBS at 1:1000 vol/vol and ultrasonicated for 30 min to redisperse the single particle, followed by centrifuging at 2000 rpm to remove aggregates. 20 µL of nanoparticle solution was injected onto the positive‐charge modified sensor chips, and images were recorded for 2 min at 100 fps. Each experiment was repeated in triplicate. The statistical histograms of silica nanoparticles were fitted with a Gaussian function to determine the peak intensity values (mean ± SD, *n* > 150) (Figure S3). The calibration curve was plotted as the intensity versus the diameter of silica nanoparticles (Figure 2f). Compensation was made for the difference in refractive index between silica and EVPs (Table S2).

### 2.5 Cell culture

The human lung cancer cell line (A549, ATCC), hepatoma cell line (HepG2, ATCC), breast cancer cell line (MCF‐7, ATCC), and normal liver cell line (L‐02, ATCC) were cultured using high‐glucose Dulbecco's modified Eagle's medium (DMEM) (Hyclone) with 10% extra‐cellular‐vesicle‐free fetal bovine serum (EV‐free FBS) (SeraPro) and 1% penicillin‐streptomycin (Gibco). The prostate cancer cell line (LNCaP, ATCC) was cultured in Roswell Park Memorial Institute 1640 (PRMI‐1640) (Hy‐clone) with 10% EV‐free FBS (SeraPro) and 1% penicillin‐streptomycin (Gibco). Cells were cultured at 37°C with 5% CO_2_ in a humidified incubator (Thermo Fisher Scientific). The cells were incubated in the T75 flask (Corning) with 30% confluent and the culture medium was collected after 48 h of culture when the cells were 70%−80% confluent.

### 2.6 EVP isolation from cell lines

EVPs were isolated based on differential centrifugation. Cell culture media (300 mL) was first centrifuged at 500 *g* for 5 min, followed by centrifugation at 2000 *g* for 45 min to remove cells. The treated medium was centrifuged at 10,000 *g* for 60 min. Then the supernatant was filtrated by 0.22 µm membrane filtration (Millipore). Finally, the filtrate was ultra‐centrifuged at 100,000 *g* for 120 min. The EVPs were washed with 50 mL 1×phosphate buffer saline (PBS, Hy‐clone), followed by ultracentrifugation at 100,000 *g* for 120 min. The purified small EVs were resuspended in 50 µL 1×PBS. In this work, we did not distinguish extracellular vesicles (EVs) from extracellular particles (EPs) due to the limitations of current isolation technology. Thus, these samples are referred to as EVPs including EVs and EPs, such as exosomere.

### 2.7 Isolation of EVPs from human plasma

This study was approved by the ethic committee of the Shanghai Jiao Tong University Affiliated Sixth People's Hospital. Written informed consent was obtained from the volunteers. Fifty‐five human plasma samples were collected (11 of breast cancer, nine of lung cancer, 11 of liver cancer, 12 of prostate cancer, and 12 of healthy donors). The patients are diagnosed with cancer by tissue biopsy. Total volumes of 200 µL of clinical plasma samples were first diluted five times with 1 × PBS, (Hyclone), and then centrifuged at 2000 *g* for 45 min to remove cells and large proteins. The treated plasma was centrifuged at 10,000 *g* for 60 min. Then the supernatant was filtrated by 0.22 µm membrane filtration (Millipore). Finally, the filtrate was ultra‐centrifuged at 100,000 *g* for 120 min. The EVPs were washed with 50 mL 1× PBS (Hyclone), followed by ultra‐centrifugation at 100,000 *g* for 120 min. The purified EVPs were resuspended in 50 µL 1×PBS. The EVP samples were stored at −80°C before use.

### 2.8 NTA analysis

The size distribution of EVP samples was characterized by NTA (Particle tracking analyser; Particle Metrix, PMX). All samples were diluted using PBS to ∼10^7^ particles/mL before measurements. The data of size distribution were analysed with NTA software. The final concentration of the EVP sample is adjusted with a dilution factor that varies among different samples. The measurements were conducted at 25°C.

### 2.9 TEM

Total of 10 µL EVP sample (∼10^12^ particles mL) were directly absorbed on Formvar/carbon‐coated copper grids for 2 mins. After blotting residual samples with filter paper, 10 µL 1% phosphotungstic acid was dropped to stain EVPs for 45 s, followed by blotting the phosphotungstic acid with filter paper. After drying at room temperature, the grids containing EVPs were observed on the Tecnai G2 spirit Biotwin TEM (FEI) at 80 kV. For immunogold labelling of EVP samples derived from LNCaP, CD63 aptamer‐conjugated gold nanoparticles were incubated with EVP samples for 30 min at 4°C, and the samples were dropped in grids for TEM detection. Total of 10 µL CD63 aptamer (1 µM) was mixed with 100 µL gold nanoparticles (4–10 nm, 2 mg/mL) at 4°C for 12 h to prepare CD63 aptamer‐conjugated gold nanoparticles, followed by blocking active sites with BSA solution (1% w/v).

### 2.10 Immunoblot analysis of EVP samples

Isolated EVP samples and cells were treated with radio immunoprecipitation assay (RIPA) lysis buffer, including protease inhibitors (Beyotime), in an ice bath for 30 min, followed by quantification with a BCA assay. Protein lysates were separated by sodium dodecyl sulfate‐polyacrylamide gel electrophoresis (SDS‐PAGE), followed by transfer to the polyvinylidene fluoride (PVDF) membrane. The transferred PVDF membranes were blocked using 5% non‐fat dry milk in TBST buffer (TBS powder, Servicebio, 0.05% Tween‐20) at room temperature for 1 h. Then blocked membranes were immunoblotted with a panel of primary antibodies including anti‐Alix (Santa Cruz, sc‐7964), anti‐Tsg101 (Santa Cruz, sc‐7964), anti‐Calnexin (Abcam, ab133615), anti‐CD63 (NOVUS, NBP2‐4225B), anti‐PTK7 (BBI, D199285‐0100), anti‐PSMA (Abcam, ab79542), anti‐HER‐2 (Abcam, ab134190), anti‐EpCAM (BBI, D263391) overnight at 4°C. Following by incubation with the corresponding HRP‐conjugated secondary antibody for 1 h at 37°C, the membranes were washed three times for 10 min at room temperature with TBST buffer. Finally, the western blot images were recorded on a gel image system (Tanon).

### 2.11 Enzyme‐linked immunosorbent assay (ELISA)

ELISA was performed to detect CD63, EpCAM, HER2, PSMA and PTK7 of EVP derived from five cell lines. First, 100 µL EVP sample was added to CD63 antibody‐modified ELISA plates (96 wells, Cusabio, China) and incubated at 37°C for 2 h. Samples were then removed and washed by washing buffer (Cusabio, China) three times. 100 µL biotin‐conjugated CD63 antibody was added to each well, followed by incubation for 1 h at 37°C. The CD63 antibody was removed, and each well was washed by washing buffer for five times, followed by adding 100 µL HRP‐avidin and incubating at 37°C for 1 h. Then, each well was washed by washing buffer five times. 50 µL tetramethylbenzidine (Cusabio, China) and stopped with stopping buffer (Cusabio, China). The plates were read at 450 nm with a microplate reader (Synergy H1, BioTek, USA).

### 2.12 Data analysis

The expression levels of target markers were defined by normalizing the target‐associated number recorded to the total number of EVPs. The total number of EVPs was measured by counting the number of EVPs binding to the positively charged sensor surfaces. The protein levels of total EVPs and three EVP subtypes were normalized by subtracting the 2.5th percentile value and dividing by (97.5th percentile value−2.5th percentile value). These normalized data were directly used for LDA‐based classification. Z‐scores were calculated by protein levels subtracted by mean and divided by row standard deviation. The significance of the difference between the EVPs from cancer cell lines and the normal cell line using individual protein markers was calculated using a two‐tailed, heteroscedastic *t*‐test (Figure S8c). The significance of the difference between the EVPs from five cell lines using individual protein markers was calculated using a two‐tailed, heteroscedastic *t*‐test (Figure S8d).

### 2.13 The machine learning algorithm

The machine learning algorithm developed to optimize sizing groups is implemented in Python 3.7. The data between 30 and 160 nm was pre‐separated into 13 subgroups at an interval of 10 nm, and a hill‐climbing algorithm was developed to find the optimal binning strategy to improve accuracy in cancer classification. Basically, the hill‐climbing algorithm includes the following steps: (1) generate a random binning result as the starting point; (2) perform the LDA classification and evaluate the accuracy; (3) generate a new binning result with the greedy strategy; (4) repeat steps (2) and (3) until a preset accuracy is achieved or after a certain number of iterations.

## 3 RESULTS AND DISCUSSION

### 3.1 The overview of SEVEN

The principle of SEVEN is based on our previous work to image, size and digitally count single small EV by interferometric plasmonic microscopy (iPM). (Yang et al., 2018, 2020) For each protein biomarker detection, SEVEN dynamically measures the size of each small EVs specifically binding onto an aptamer‐coated sensor surface and divides them into size‐dependent subpopulations (Figure 1a). Corresponding size‐dependent binding curves are then constructed by digital counting of EVPs, from which parameters including the maximum binding number and the exponential coefficient are quantified. For EVPs from different cell sources measured on different aptamer‐coated sensor surfaces, these parameters form a multidimensional matrix containing the correlative information between sizes and protein biomarkers (Figure 1b). For EVPs from cancerous cells and healthy cells, the multidimensional data matrix is fed into a linear discriminant analysis (LDA) model to determine the cell sources (Figure 1c).

### 3.2 Verification of EVP sample

EVP samples were collected from both cell lines and the plasma of cancer patients. Five different cell lines, including A549 (lung cancer), HepG2 (liver cancer), MCF‐7 (breast cancer), LNCaP (prostatic cancer) and L‐02 (normal liver cells), were cultured to derive EVPs. EVPs were isolated using ultracentrifugation methods as described previously (Thery et al., 2006). The sizes of isolated EVs were below 200 nm as measured by nanoparticle tracking analysis (NTA) (Figure 2a and Figure S1), and the original concentration varied from 10^10^/mL to 10^12^/mL adjusted with a dilution factor. The morphology of EVPs was characterized by transmission electron microscopy (TEM), showing typical entire and saucer‐like shapes (Figure 2b). According to the guidelines in minimal information for studies of extracellular vesicles 2018 (MISEV 2018) (Thery et al., 2018), we found that three universal EV‐positive plasma membrane proteins, Alix, CD63 and Tsg101 were positively expressed, and one EV‐negative protein, Calnexin, was negatively expressed with western blot (Figure 2c).

### 3.3 Plasmonic imaging and detection of EVPs

In SEVEN, the iPM system offers a unique approach to imaging and characterizing single EVP. After binding to the chips, EVPs showed an intact morphology as characterized by scanning electron microscope (SEM) (Figure S2). We first established the calibration curve between iPM intensity and particle sizes using silica nanoparticles (Figure 2d and Figure S3), and measured the sizes of EVP by intercalation after compensating for the difference in refractive index between EVP and silica (Table S2). The comparison between the iPM images and atomic force microscopy (AFM) images of the same particles validated the capability for single particle analysis (Figure S4). Using EVPs samples from MCF‐7 as an example, the number of EVPs binding onto optimized CD63‐aptamer‐modified sensor surfaces (Figure S5) within 15 min correlated well with the concentrations of total EVPs in the range from 5 × 10^6^/mL to 5 × 10^9^/mL (Figure 2e). The specificity of measuring CD63‐positive EVPs was verified by comparing the binding on bull serum albumin (BSA)‐coated surfaces and a dissociation experiment (Figure S6). Note that the conventional enzyme‐linked immunosorbent assay (ELISA) failed to measure the CD63‐positive EVPs.

Besides measuring the size and concentration, SEVEN could also report the average expression intensity level of specific proteins in EVP samples. For EVP samples of the five cell lines at a concentration of 2 × 10^10^/mL, we compared the CD63 expression levels measured by ELISA with the percentage of CD63‐positive EVPs in total EVPs determined by iPM, which showed a good linear correlation (R^2^ > 0.99, Figure 2f). We note that the protein levels reported by SEVEN are not the expression level on single EVP but are related to the total proteins from all EVPs.

### 3.4 Limitations in bulk analysis

We first demonstrated the capability of SEVEN in profiling proteins in the total EVP population for bulk analysis to determine cancer type, which is a common practice. Seventy‐two EVP samples were collected from the cancer cell lines of A549, HepG2, MCF‐7 and LNCaP, and 18 samples were collected from L‐02 healthy liver cell lines as the control. The expression of the five biomarkers was first confirmed by Western blot (Figure 2c and Figure S7). A panel of membrane protein markers was selected, including CD63, EpCAM, HER2, PSMA and PTK7 (Supplementary Text). Expression levels of CD63, EpCAM, HER2, PSMA and PTK7 in the EVP samples from the five cell lines were measured as the percentage of target‐positive EVPs in total EVPs (Figure S8a). The heterogeneity among different batches of EVP samples was obvious in both iPM and ELISA assays (Figure 2g and Figure S8b), which is inevitable due to the intrinsic variations in cell conditions and other environmental factors. The exponential coefficients related to the association rates were quantified, which were also heterogeneous among different cell lines (Figure 2h).

**FIGURE 3:**
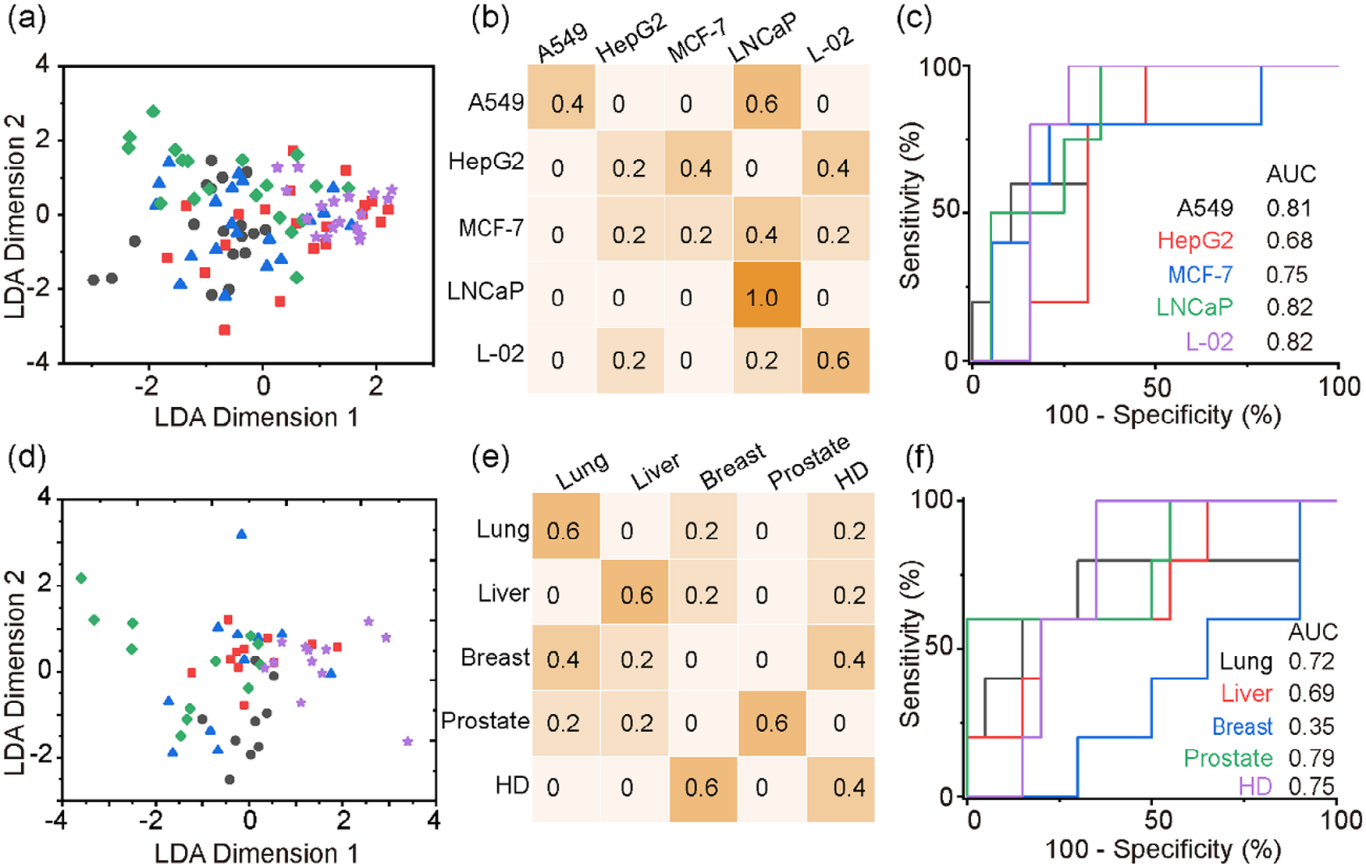
Cancer classification by bulk analysis of surface protein markers of EVP from cell lines (a–c) and clinical plasma samples (d–f). (a, d) LDA classification by bulk analysis with five protein biomarkers from total EVP. (b, e) Probability matrix summarizing the classification results. (c, f) The receiver operating characteristic (ROC) plots.

EVPs from the four cancer cell lines showed significantly higher protein levels than the healthy control from L‐02 (*p* < 0.01, Figure S8c), indicating the potential to perform cancer diagnosis with these biomarkers. But when performing the cancer classification with the expression level and exponential coefficient of only one of the biomarkers, none was able to classify all five cancers with *p* < 0.05 (Figure S8d) in a pairwise comparison. Even for the well‐recognized specific markers, such as HER2 or PSMA, it was not able to distinguish breast cancer from prostate cancer by a single marker. When the expression levels and exponential coefficients of all five biomarkers were analysed with the LDA model (Tian et al., 2021), the average classification accuracy was only 45% for the 72 samples (Figure 3a,b). The areas under the curve (AUC) were 0.81, 0.68, 0.75, 0.82 and 0.82 respectively, using receiver operating characteristic (ROC) analysis (Figure 3c). For comparison, the classification accuracy using ELISA was found to be 56% for 25 samples, which corresponds well with the results of SEVEN for 25 samples, between 12% and 72% (Figure S8e,f).

These results from cell models correspond well with the clinical tests. We collected 55 plasma samples, including 43 plasma samples from cancer patients (nine for lung cancer, 11 for liver cancer, 11 for breast cancer and 12 for prostate cancer) and 12 samples from healthy donors. In a similar bulk analysis, the average accuracy in determining cancer type by SEVEN was 43% (Figure 3d,e) with an AUC of 0.72, 0.69, 0.35, 0.79 and 0.75 respectively (Figure 3f). These results suggest that bulk analysis with the mixed EVP population could lead to poor accuracy in cancer diagnosis. One of the reasons could be due to the heterogeneity at the single EVP level. For example, when examining the CD63 level in EVP from LNCaP cells using immunoelectron microscopy, different numbers of immunogold nanoparticles were observed on EVPs (Figure S9). We then investigated the possibility of improving cancer diagnosis accuracy by exploring the protein heterogeneity at the subpopulation level.

### 3.5 Correlation between membrane proteins and sizes

**FIGURE 4:**
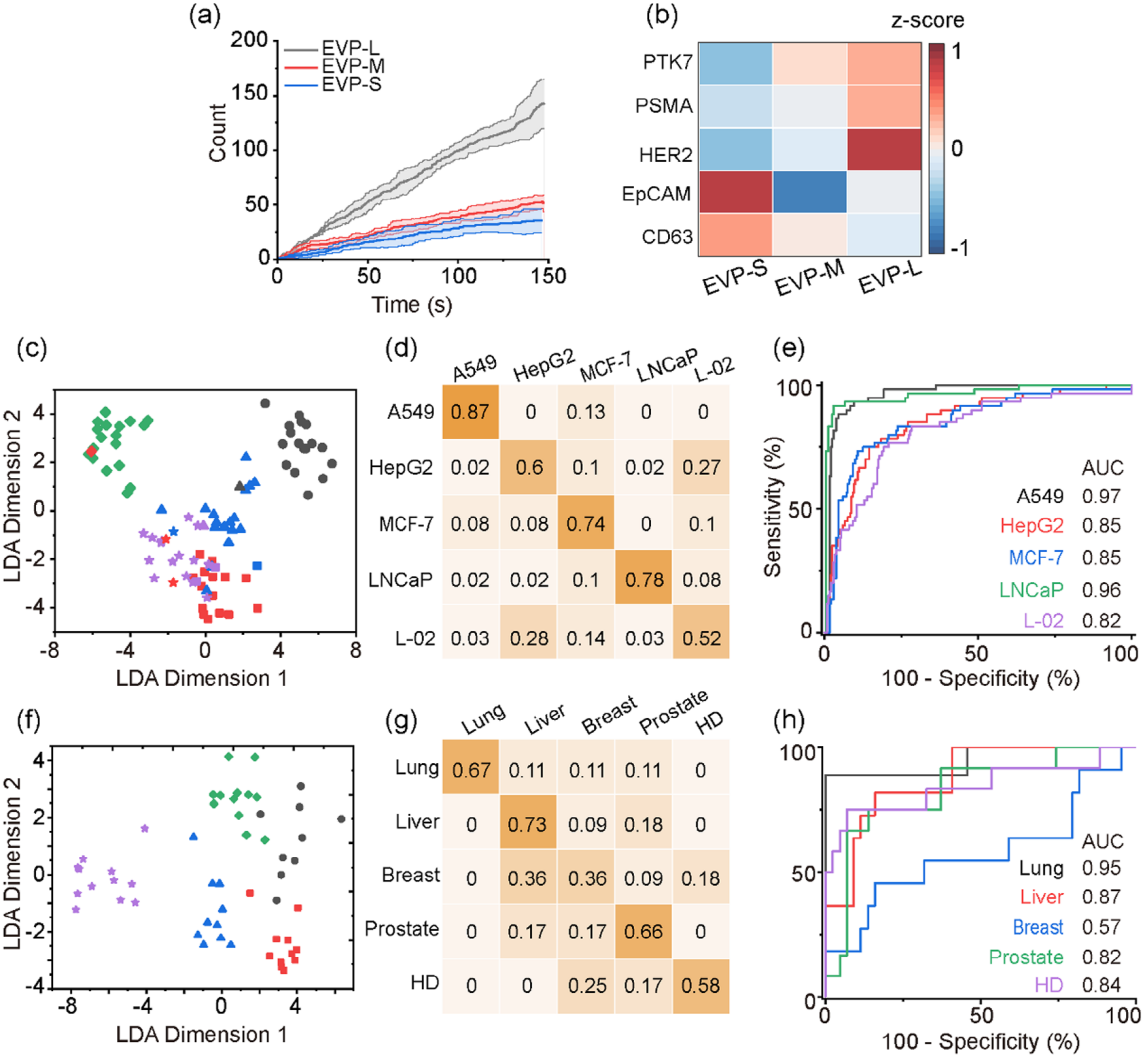
Correlation between membrane proteins and sizes as EVP signatures for cancer classification by groupi‐ng EVPs into three subpopulations in (c–e) cell lines and (f–h) clinical plasma samples. (a) Typical binding curves of LNCaP‐derived EVP subpopulations on PSMA‐modified chips. (b) Heatmap illustration of the relative abundance of EVP (A549) markers in EVP‐S, EVP‐M and EVP‐L. (c, f) The LDA classification results, (d, g) probability matrix and (e, h) the ROC plots.

For simplicity, the EVPs were first empirically divided into three size‐dependent subpopulations, including the EVP‐S (30–70 nm), EVP‐M (70–120 nm) and EVP‐L (120–160 nm). With the single EVP imaging and sizing capability of iPM, the binding curves of three subpopulations were digitally plotted (Figure 4a and Figure S10). The exponential coefficients were obviously heterogeneous. For example, the exponential coefficients were 0.0070, 0.0091 and 0.0089 s for EVP‐S, EVP‐M and EVP‐L binding with PSMA‐aptamer, and 0.0013, 0.0015 and 0.0028 s with CD63‐aptamer respectively, and LNCaP and L‐02‐derived small EVs showed much faster binding rates than A549‐derived EVPs.

We further calculate the *z* score of protein levels from the three subpopulations to highlight the difference (Zhang et al., 2018) (Figure 4b and Figure S11). In EVPs from all cell lines other than A549, all protein markers were found to enrich in EVP‐L. For A549, EpCAM and CD63 were mainly expressed in the EVP‐S subpopulations, but HER2, PSMA and PTK7 were expressed in the EVP‐L. This confirms that the selective set of membrane protein expression levels in EVPs is not directly proportional to membrane areas, but instead, they might be assembled purposefully. The reason why specifically only EpCAM and CD63 from A549 cells were highly expressed in EVP‐S is still unknown. Note that the EVP‐S population weighed only a small portion of the total EVPs (Figure 2a). Thus, when analysing EpCAM and CD63 from the total EVPs, the majority as EVP‐M and EVP‐L would give a large background noise, which could explain the poor accuracy in bulk analysis.

The multidimensional information matrixes of EVPs, including the protein levels and association rates of the three subpopulations, were input into a LDA model to discriminate between the five cell lines. For cell models, the average classification accuracy reached 70% (Figure 4c,d), with the AUC of 0.97, 0.85, 0.85, 0.97 and 0.82 for the five cell lines, respectively (Figure 4e). For clinical samples, the average classification accuracy increased from 43% to 60%, with the AUC of 0.95, 0.87, 0.57, 0.82, 0.84 for lung cancer, liver cancer, breast cancer, prostate cancer and healthy donors (Figure 4f–h). Although the accuracy is not yet enough for clinical applications, the improvement by using the correlation information is convincing.

### 3.6 Optimizing the subgrouping strategy

Simply dividing the EVP into small, medium, and large subsets is intuitive but brutal, both in previous work by isolating the subsets (Zhang et al., 2018) and in this work by empirically setting thresholds. There has been little evidence showing why the membrane proteins and contents should be packed in such a simple size‐dependent manner. This could be one of the reasons that the accuracy was only improved to 60% in the above clinical tests. A deeper study is hindered due to the lack of technologies to isolate the EVP subsets within narrower size ranges. However, in SEVEN, the iPM system offers a sizing accuracy of 10 nm, which provides the opportunity to potentially address this problem by setting more groups with smaller size ranges.

**FIGURE 5:**
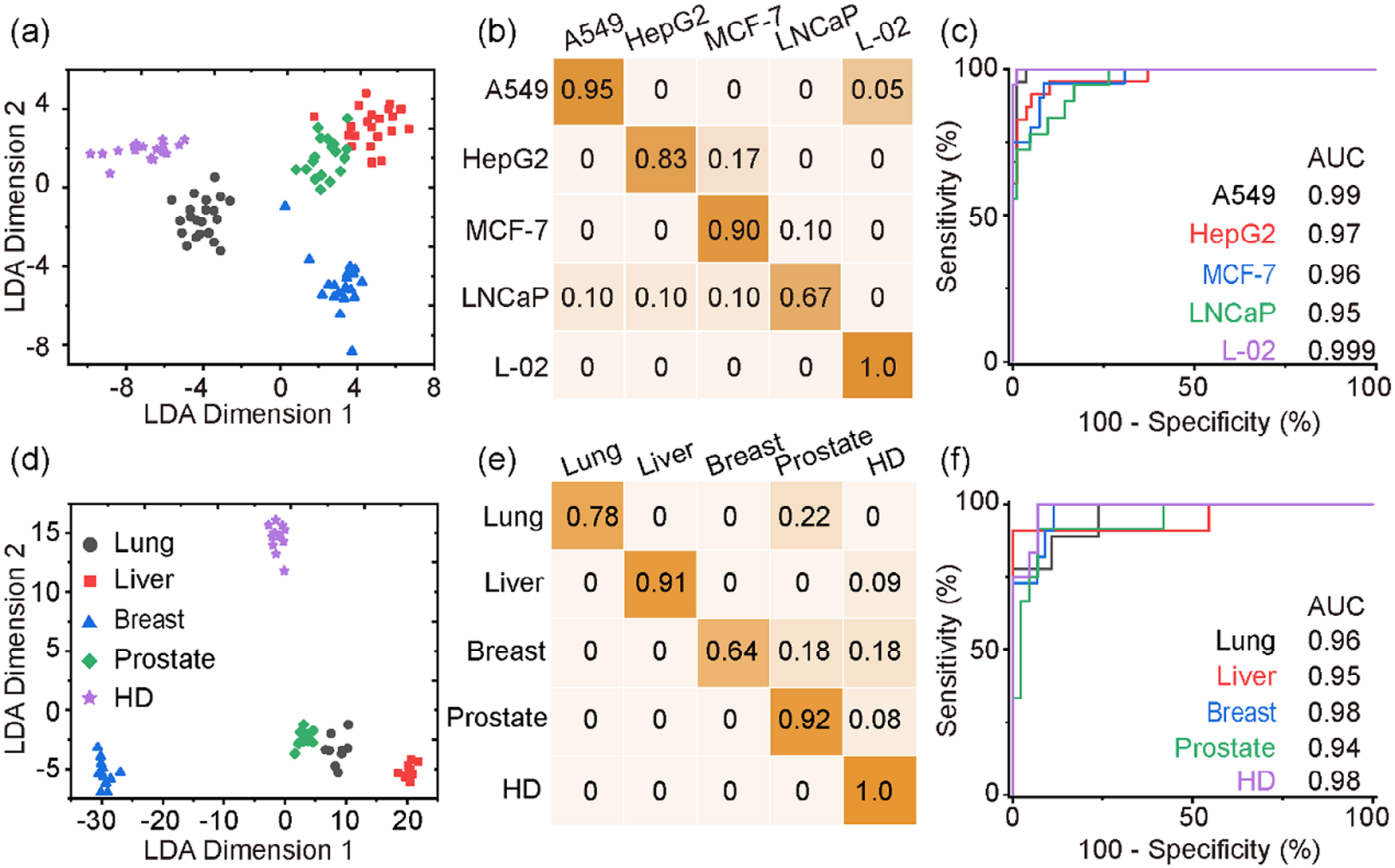
Correlation between membrane proteins and sizes as EVP signatures for cancer classification by subgrouping EVPs into multiple subpopulations with a 10 nm size interval in (a–c) cell lines and (d–f) clinical plasma samples. (a, d) The LDA classification results, (b, e) probability matrix and (c, g) ROC plots.

We re‐analysed the data measured by SEVEN between 30 and 160 nm as 13 subgroups at an interval of 10 nm. Instead of empirically combining some of the sub‐groups, we developed an artificially intelligent algorithm to automatically search for the optimal size partition to achieve the best classification accuracy (Figure S12). The results show that when the EVP were grouped by sizes within 30–40, 40–70, 70–100, 100–110, 110–130, 130–140 and 140–160 nm, the average classification accuracy for cell models dramatically increased to 87% (Figure 5a,b), with the AUC of 0.99, 0.97, 0.96, 0.95 and 0.999 for the five cell lines, respectively (Figure 5c). Besides, the healthy liver cell line L‐02 was fully separated from cancerous cell lines. Similarly, for clinical tests, the optimal classification accuracy was achieved at 85% when grouping the EVP subsets as 30–40, 40–50, 50–60, 60–70, 70–80, 80–90, 90–110 and 110–160 nm (Figure 5d,e). The AUC were 0.96, 0.95, 0.97, 0.94 and 0.98 for lung cancer, liver cancer, breast cancer, prostate cancer and healthy donors (Figure 5f).

The accuracy achieved at 85% is among the best performances reported (Liu et al., 2019). However, the true meaning of the grouping outcome is unknown. In fact, it is still unknown why cells would pack the selective set of membrane proteins of EVP in a size‐dependent manner. It is worth noting that overfitting might happen during the optimization, which should be further validated in the future with a much larger number of samples. However, these results indicate that the packing of the selective set of membrane proteins onto EVs of different sizes could be highly heterogeneous, and linking the size to membrane proteins could help improve the cancer classification accuracy.

### 3.7 Limitations and perspective

The presented approach has enabled us to perform the very first experimental study using the correlation between membrane proteins and EVP sizes as a signature for cancer diagnosis. But despite these unique capabilities, it suffers from a few limitations, especially in the throughput. In each channel, only the correlation between one type of membrane protein and EVP sizes in one EVP sample could be obtained. This could hamper the application in clinical settings, and thus require further improvements in system automation and sensor array design. We chose a panel including five membrane proteins as an example that successfully validated the effectiveness of using the correlation between membrane proteins and EVP sizes as a signature for cancer diagnosis. We note that while proteomic study is useful for discovering new molecular markers, clinical diagnosis typically uses a panel of only a few markers due to the limitations in throughput and cost.

We note that there are several existing techniques capable of measuring membrane proteins and sizes of EVPs, such as the NanoFlow (Tian et al., 2018), NTA (Shao et al., 2018) and the ExoView (Daaboul et al., 2016). However, we present here the first attempt to explore the correlation between membrane proteins with sizes as an EVP signature for cancer classification. This may mainly benefit from the high sensitivity of the iPM system to size single EVP down to 30 nm and from the ability to quantify membrane protein expression level and binding kinetics. Additionally, in this work, the influence of EVP‐free proteins could be ruled out as we performed the digital detection of EVPs. EVP‐free proteins could not be identified in the image, and thus only EVP‐bound markers were included in the analysis. This is one of the advantages of using the SEVEN platform as compared with non‐imaging approaches. We anticipate that the combination of these techniques to quantify multiple EVP protein markers and sizes simultaneously could further improve the clinical testing accuracy.

## 4 CONCLUSION

We have presented the single EVP enumerating (SEVEN) approach to explore the in‐depth correlation information between membrane proteins and sizes. Benefiting from the single particle imaging and detection capability of interferometric plasmonic microscopy, SEVEN circumvents the challenges of precise isolation of subpopulations. Studies on five cell lines and five protein biomarkers have provided new evidence in the correlation between the protein levels and the size distribution of EVPs. Experiments with EVPs derived from both cancer cell lines and clinical plasma samples show that SEVEN could effectively improve the classification accuracy over bulk analysis. This work has thus highlighted the importance of exploring the underlying relationship between different dimensions of heterogeneity of EVPs for developing better diagnostic performance. With further improvement in throughput and system automation, we anticipate that SEVEN could find clinical applications for cancer diagnosis.

## AUTHOR CONTRIBUTIONS

Hui Yu and Yuting Yang conceived the idea; Chunhui Zhai, Jiaying Xu and Yuting Yang performed the experiments; Chunhui Zhai and Feng Xie analysed data; Weiqiang Zheng helped with instrumentation; Haiyan Hu. and Xianting Ding provided samples and helped with discussion; Chunhui Zhai and Hui Yu wrote the paper.

## CONFLICT OF INTEREST STATEMENT

The authors declare no competing interest.

## Supporting information

Supporting Information
