# Supplementary material for: Correlation between membrane proteins and sizes of extracellular vesicles and particles: A potential signature for cancer diagnosis
Source: J Extracell Vesicles. 2023 Dec 5;12(12):12391. doi: 10.1002/jev2.12391 (PMC10696525; doi:10.1002/jev2.12391)
Supplement: Supplementary file 1 — Supporting Information [file JEV2-12-12391-s001.docx]

Supporting Information for

**Correlation between membrane proteins and sizes of extracellular vesicles and particles: a potential signature for cancer diagnosis**

*Chunhui Zhai^1^, Feng Xie^1^, Jiaying Xu^1^, Yuting Yang^2^*, Weiqiang Zheng^2^, Haiyan Hu^3*^, Xianting Ding^1^*, Hui Yu^1^**

^1^ School of Biomedical Engineering, Shanghai Jiao Tong University, Shanghai, 200030, People’s Republic of China

^2^ Department of Instrument Science and Engineering, School of Electronic Information and Electrical Engineering Shanghai, 200030, People’s Republic of China

^3^ Oncology Department, Shanghai Jiao Tong University Affiliated Sixth People’s Hospital, Shanghai, 200233, People’s Republic of China

*Corresponding author. E-mail:

Prof. Hui Yu (E-mail: hui.yu@sjtu.edu.cn)

Prof. Xianting Ding (E-mail: dingxianting@sjtu.edu.cn)

Prof. Yuting Yang (E-mail: yuting.bme@sjtu.edu.cn)

Prof. Haiyan Hu (E-mail: xuri1104@163.com)

**This PDF file includes:**

Supplementary text

Supplementary Figures S1 to S12

Supplementary Tables 1-2

Supplementary Text

**1.The surface marker selection**

CD63 is a universal exosomal biomarker, which is mostly used in exosomes immunological detection methods(1). Our western blotting results (**Figure 2b**, **Figure S7**) showed a positive expression among the all five samples, indicating many exosomes in the samples. EpCAM is overexpressed in various epithelial carcinoma, and it is widely used in exosomal detection. EVPs derived from A549, MCF-7, HepG2 and LNCaP showed positive expression of EpCAM, which is consistent with previous studies(2). In our experiments, the normal liver cell showed negative expression of EpCAM(3). HER2 is one of the ErbB family members, which is related to the occurrence and development of breast cancer as well as is an effective prognostic indicator of breast cancer. Besides, HER2 is a tyrosine kinase receptor, which is a potent cancer-related protein in various cancers such as lung (A549 cell lines)(4, 5), and prostate cancer(6, 7). The expression of HER2 was also detected in exosomes derived from liver cells, for example HepG2 and L-02(8). We also observed the expression of HER2 in the EVPs from all the five cell lines in our experiment (**Figure S7**). PSMA is a prostate cancer marker, which is highly expressed in LNCaP cell line and LNCaP EVPs(9). But PSAM has also been discovered to be expressed in non-small cell lung cancer(10), and in extracellular vesicles of MCF-7(11)and HepG2(12, 13). In the five cell lines we used in this work, PSMA is positive in all samples. PTK7 is a key transmembrane receptor protein, which plays an essential role in regulating the Wnt signaling network(14). A549, MCF-7, LNCaP cells do not express PTK7(15, 16), which is consistent with our western blot results. The other two cell lines, HepG2 and L-02 expressed PTK7(17).

**2. Supplementary Figures**


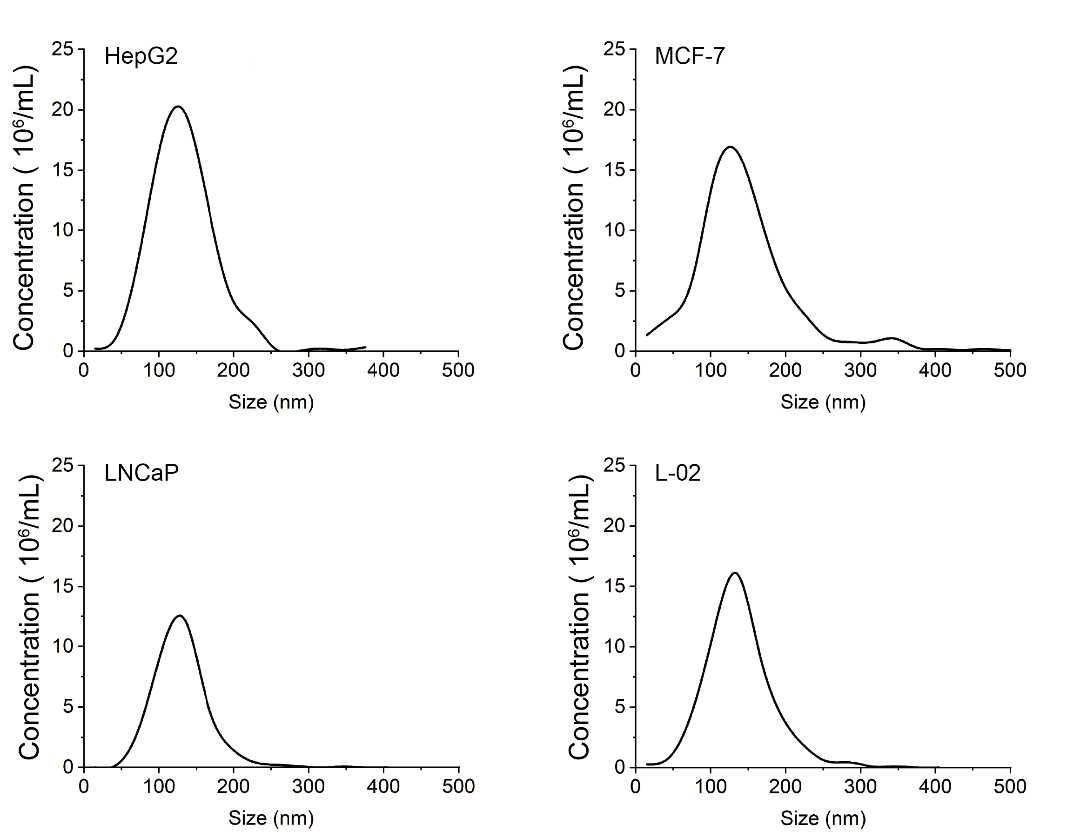


**Figure S1**. NTA measurement of HepG2, MCF-7, LNCaP and L-02 derived EVP samples


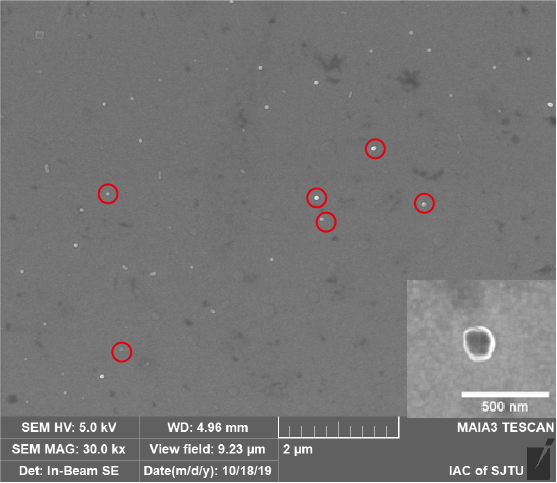


**Figure S2** TEM images of EVPs binding onto HER-2-modified chips.


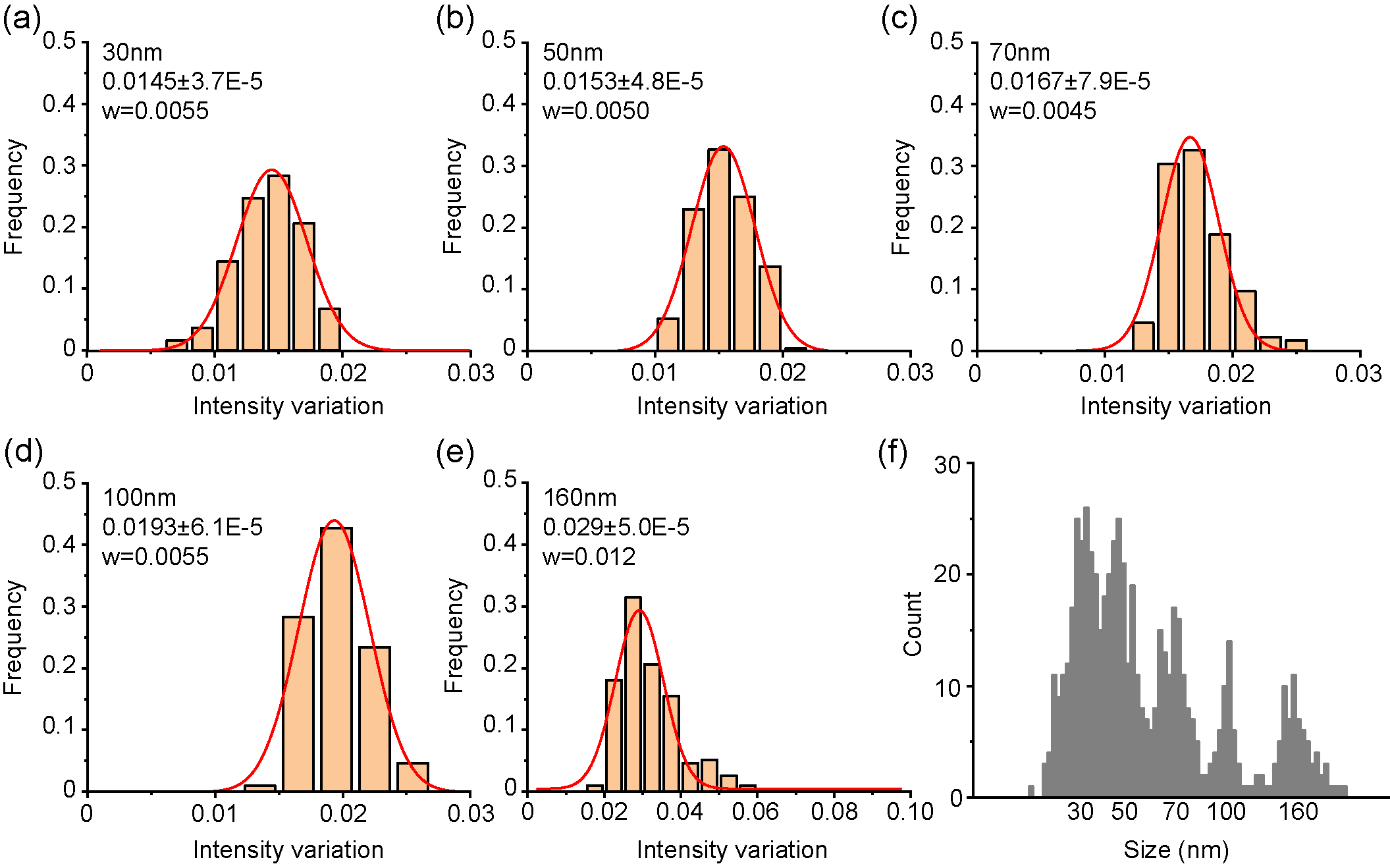


**Figure S3**. The iPM intensity change induced by single silica nanoparticles with diameters of (a) 30 nm (b), 50 nm, (c) 70 nm, (d) 100 nm and (e) 160 nm, and the size distribution of mixed SiO2 particels.


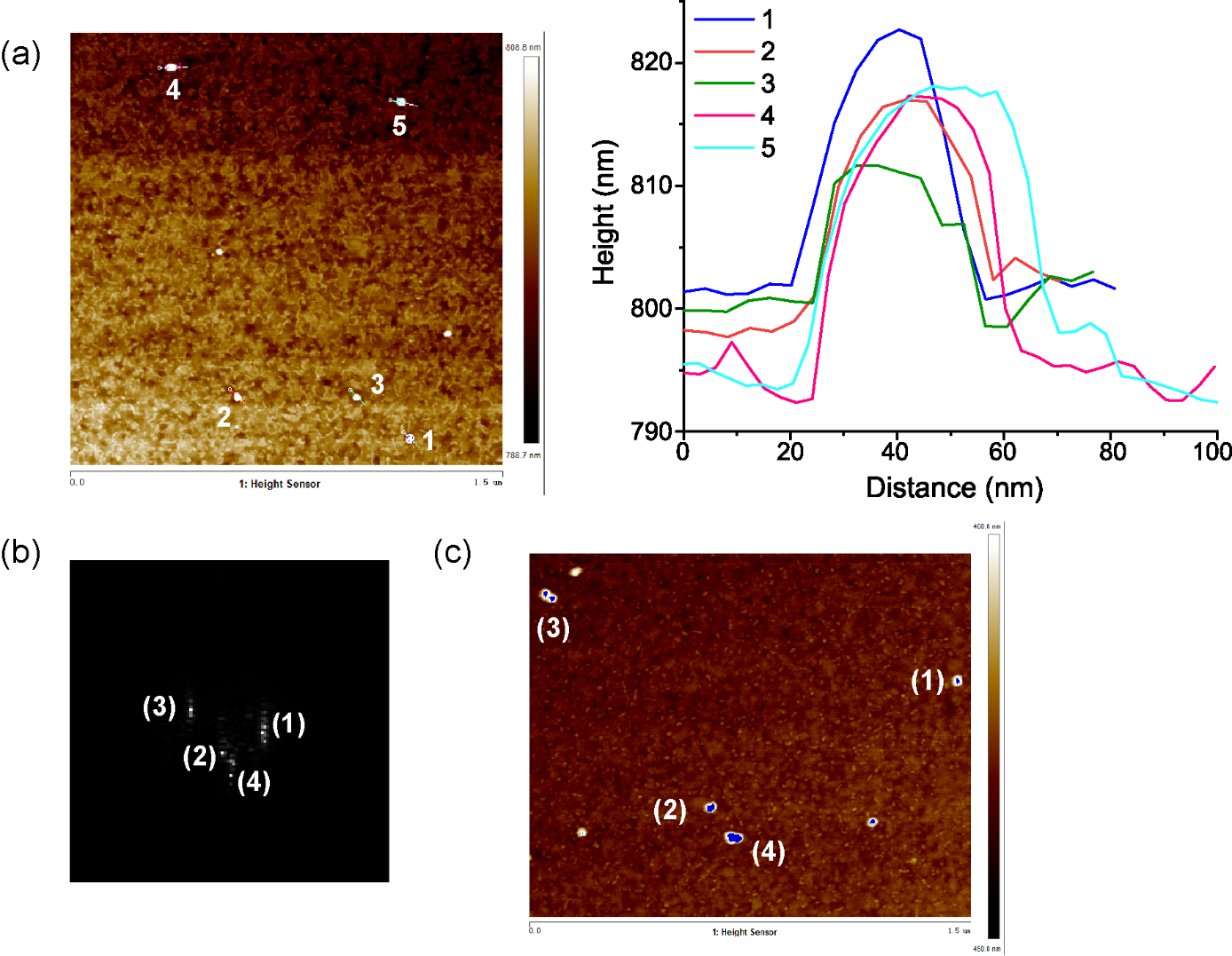


**Figure S4** AFM and iPM images of the same silica. (a)AFM images for 30 nm silica nanoparticles (left) and their height profilings (right). (b) and (c) are the iPM and AFM images for the same silicas.


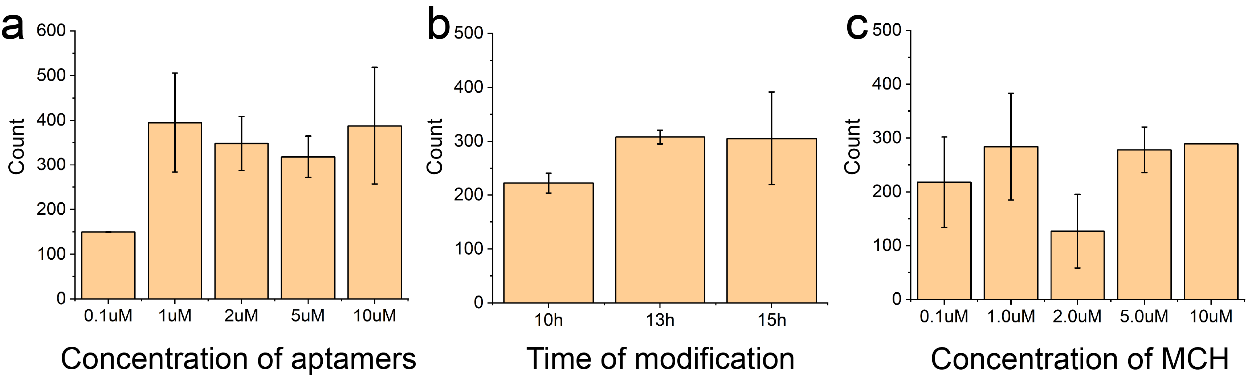


**Figure S5**. Optimization of surface modification. The recorded EVP (derived from MCF-7 cell line, 5×10^9^/mL) number vs. (a) the concentration of CD63 aptamers, (b) the time of modification, and (c) the concentration of MCH.


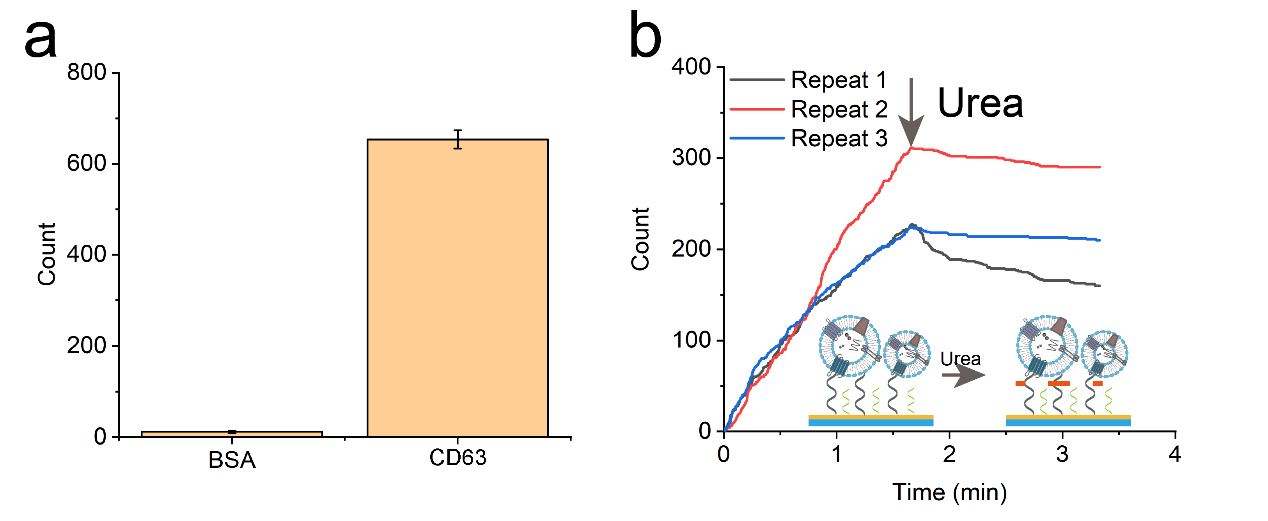


**Figure S6.** The specificity in EVP detection. (a) The number of EVPs recorded on BSA and CD63 modified sensor surface, (b) the binding curves and competing tests for with urea.


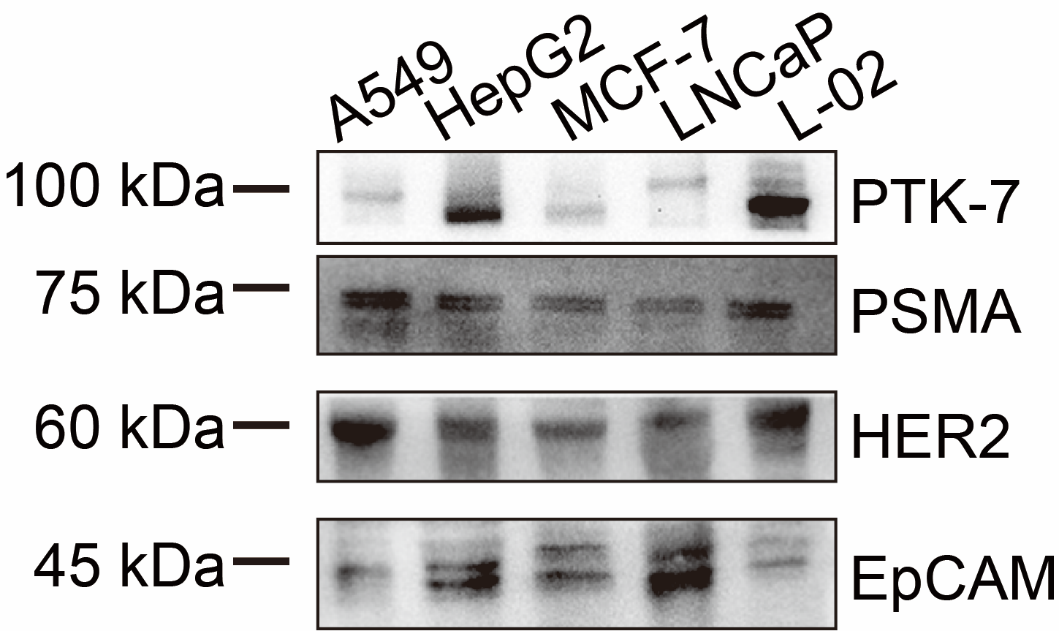


**Figure S7**. Western blot characterization of A549-, HepG2-, MCF-7-, LNCaP- and L-02- derived EVP samples.


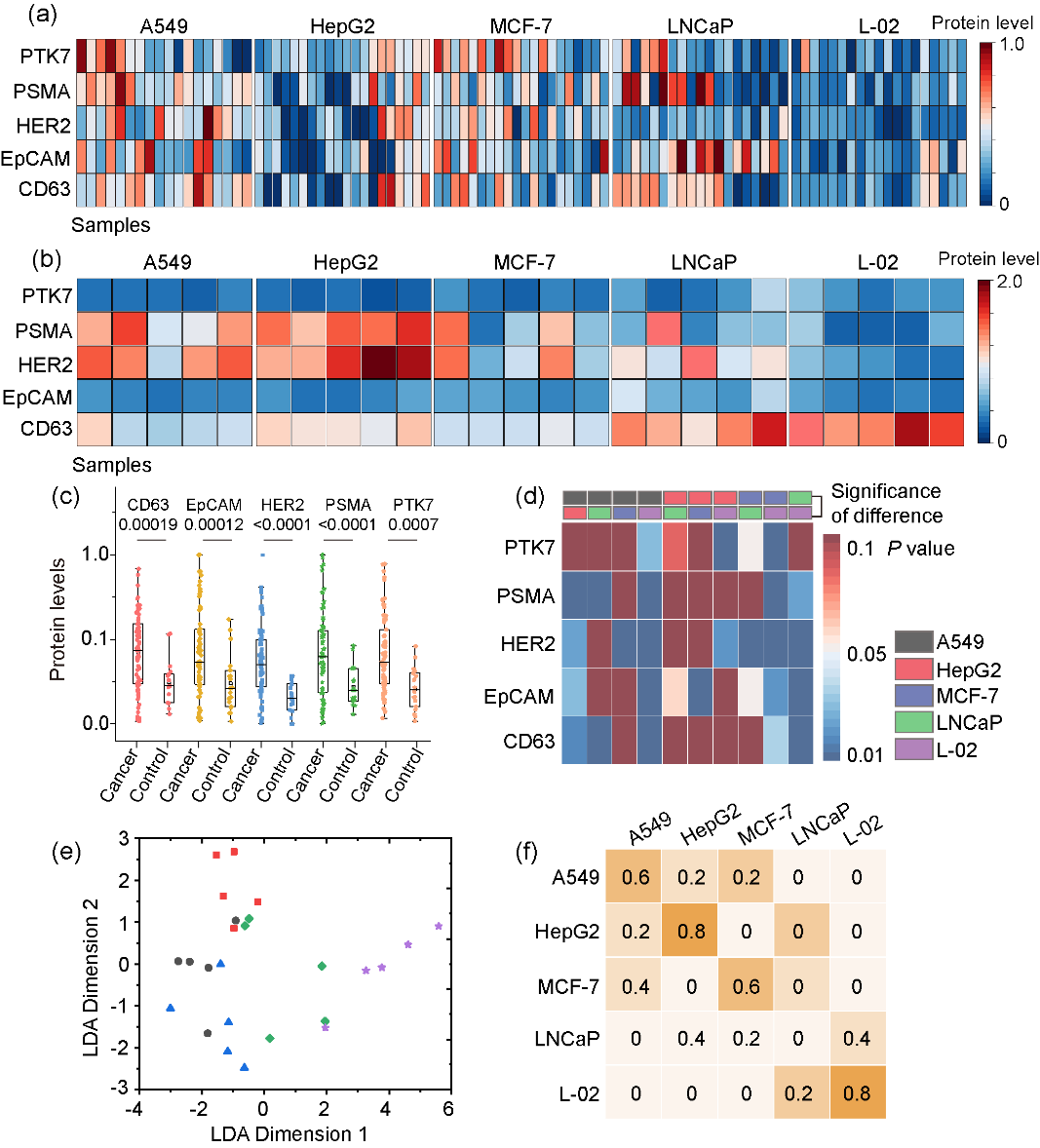


**Figure S8.** Profiling total EVP surface protein markers by SEVEN and ELISA. (a) Heat map of EVP surface protein in 5 cell lines (18 samples for each cancer cell line) by SEVEN and by ELISA (b) (5 samples for each cancer cell line). (c) Elevated protein levels of all 5 protein markers in EVPs derived from cancer cell lines (n = 78; means ± s.e.m.) than normal cell line (n = 18; means ± s.e.m.). (d) The statistical differences in distinguishing cancer types (n = 18 samples for each cell type) by a single biomarker by independent samples t-test. (e) Cancer classification using five protein biomarkers measured by ELISA of total EVPs by LDA. (f).


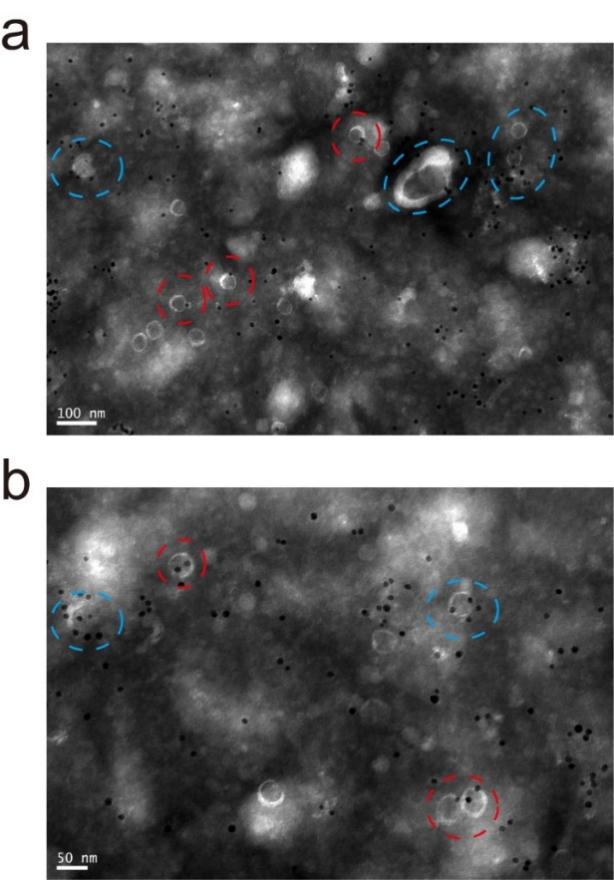


**Figure S9.** Immunoelectron microscopic image of the CD63-aptamer-coated gold nanoparticles on EVPs.


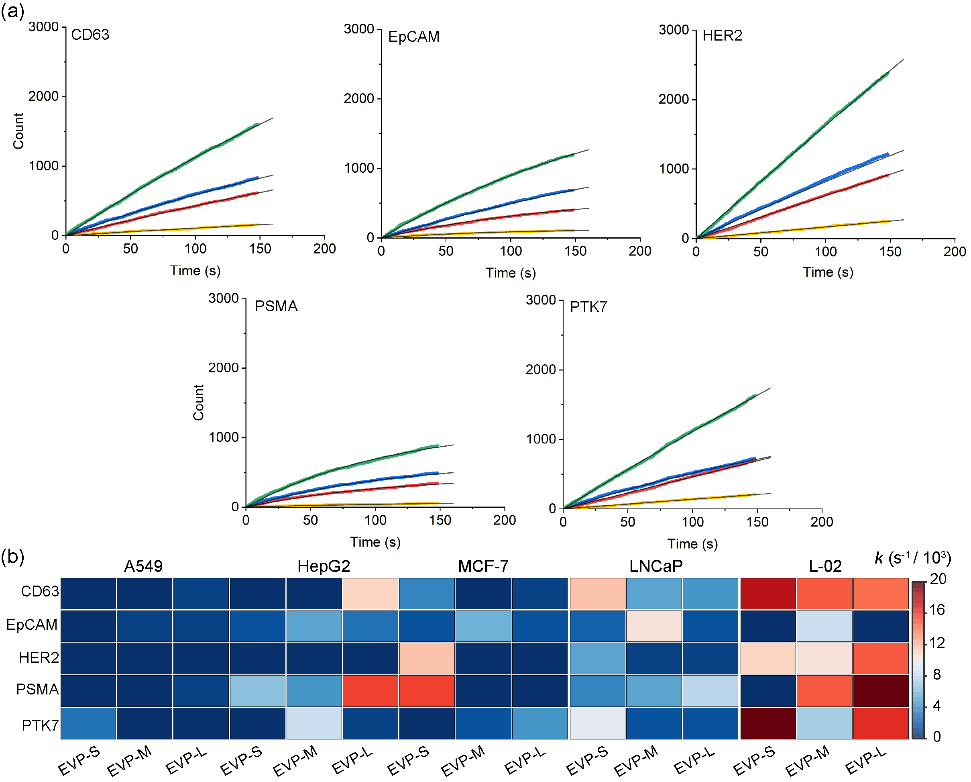


**Figure S10**. (a) The examples of binding kinetics of MCF-7-derived EVPs on aptamers modified sensor chips (Green for all EVP, blue for EVP-L, red for EVP-M and yellow for EVP-S). (b) The heat map of apparent association rate of five cell lines.


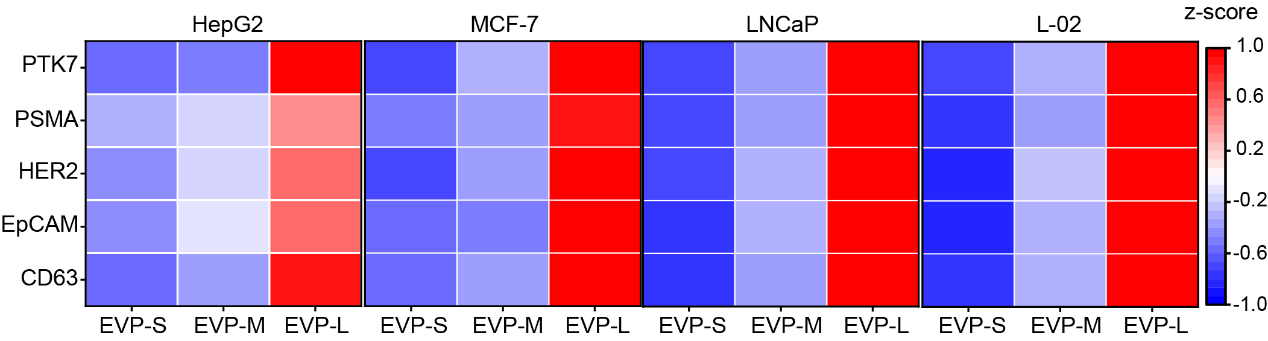


**Figure S11**. Heatmap illustration of the relative abundance of EVPs (HepG2, MCF-7, LNCaP, L-02) markers in EVP-S, EVP-M and EVP-L. Scale shown is z score subtracted by mean and divided by row standard deviation (that is, Δ (protein levels − mean)/s.d.).

**
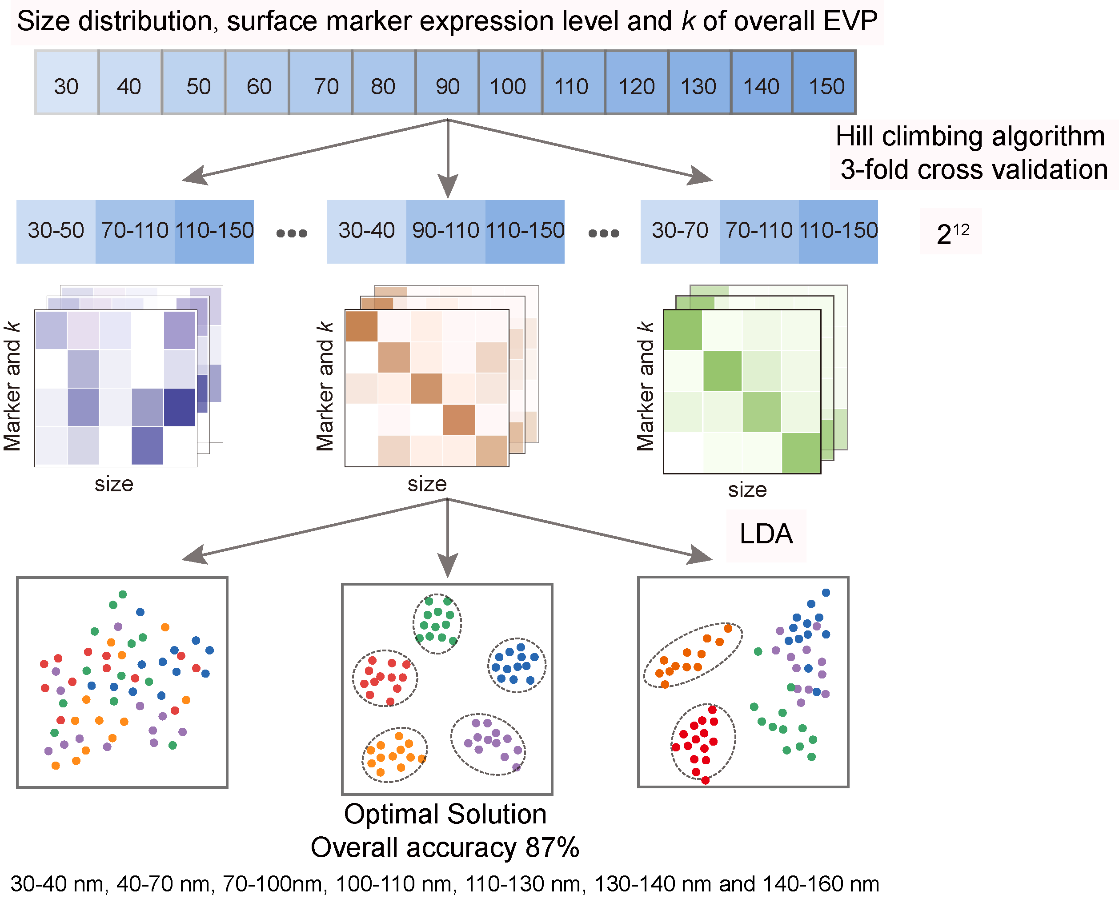
**

**Figure S12.** The schematic of Automatic Searching algorithm. The data between 30 to 160 nm were pre-separated into thirteen subgroups at the interval of 10 nm, and a hill-climbing algorithm was developed to find the optimal binning strategy to improve accuracy in cancer classification. Basically, the hill-climbing algorithm includes the following steps: 1) generate a random binning result as the starting point; 2) perform the LDA classification and evaluate the accuracy; 3) generate a new binning result with the greedy strategy; 4) repeat step 2 and 3 until a preset accuracy is achieved or after certain number of iterations.

**Table S1. Summary of aptamers(18)**

| Aptamer | Sequence (5’ to 3’) |
| --- | --- |
| HER2 | 5'-GGG CCG TCG AAC ACG AGC ATG GTG CGT GGA CCT AGG ATG ACC TGA GTA CTG TCC-3' |
| EpCAM | 5'-CAC TAC AGA GGT TGC GTC TGT CCC ACG TTG TCA TGG GGG GTT GGC CTG-3' |
| PTK7 | 5'-ATC TAA CTG CTG CGC CGC CGG GAA AAT ACT GTA CGG TTA GA-3 |
| CD63 | 5'-CAC CCC ACC TCG CTC CCG TGA CAC TAA TGC TA-3' |
|  |  |
| PSMA | 5‘-GCG TTT TCG CTT TTG CGT TTT GGG TCA TCT GCT TAC GAT AGC AAT GCT-3‘ |

**Table S2. Scattering cross section (σ)^*^ of EVP and silica**

| Refractive index | 1.38 (EVP) | 1.45 (silica) | 1.45 (silica) | 1.45 (silica) |
| --- | --- | --- | --- | --- |
| Size (nm) | 30 | 24 | 23 | 22 |
| $\sigma$ (×10^-9^ μm^2^) | 5.38 | 7.95 | 6.16 | 4.72 |
| $\sqrt{\sigma}$ (×10^-5^ μm) | 7.33 | 8.91 | 7.84 | 6.87 |

*The scattering cross sections of EVP and silica nanoparticles were determinded by the incident electric field and its polarizability ($\alpha$) , or, equivalently, cross-section $\left( \sigma\right)$ according to Raylegh scattering. The textbook formulae $\alpha=3V\left( n_{s}^{2}-n_{m}^{2} \right)\left( n_{s}^{2}+2n_{m}^{2} \right)^{-1}$ and $\sigma= \frac{8}{3}\pi^{3}\alpha^{2}{(\frac{\lambda}{n_{m}})}^{-4}$, where *V* is the object volume, $n_{s}$ its refractive index, $n_{m}$ the refractive index of the surrounding medium and $\lambda$ the illumination wavelength (19) (here, λ = 640 nm). The results showed that the $\sigma$ of a 30 nm sEVP (with refractive index 1.38) is equivalent to that of a 25 nm silica nanoparticle with refractive index 1.45.

**References**

1. N. Cheng *et al.*, Recent advances in biosensors for detecting cancer-derived exosomes. *Trends Biotechnol* **37**, 1236-1254 (2019).

2. Y. Fan *et al.*, High-sensitive and multiplex biosensing assay of NSCLC-derived exosomes via different recognition sites based on SPRi array. *Biosens Bioelectron* **154**, 112066 (2020).

3. M. Zhu *et al.*, HBx drives alpha fetoprotein expression to promote initiation of liver cancer stem cells through activating PI3K/AKT signal pathway. *Int J Cancer* **140**, 1346-1355 (2017).

4. P. A. Bunn *et al.*, Expression of Her-2/neu in human lung cancer cell lines by immunohistochemistry and fluorescence in situ hybridization and its relationship to in vitro cytotoxicity by trastuzumab and chemotherapeutic agents. *Clinical Cancer Research* **7**, 13 (2001).

5. T. Y. Rakovich *et al.*, Highly sensitive single domain antibody-quantum dot conjugates for detection of HER2 biomarker in lung and breast cancer cells. *ACS Nano* **8**, 14 (2014).

6. M. Dahl *et al.*, Sarcosine induces increase in HER2/neu expression in androgen-dependent prostate cancer cells. *Mol Biol Rep* **38**, 4237-4243 (2011).

7. R. Jahanban-Esfahlan *et al.*, The herbal medicine Melissa officinalis extract effects on gene expression of p53, Bcl-2, Her2, VEGF-A and hTERT in human lung, breast and prostate cancer cell lines. *Gene* **613**, 14-19 (2017).

8. H. Di *et al.*, Nanozyme-assisted sensitive profiling of exosomal proteins for rapid cancer diagnosis. *Theranostics* **10**, 9303-9314 (2020).

9. B. Li *et al.*, Facile fluorescent aptasensor using aggregation-induced emission luminogens for exosomal proteins profiling towards liquid biopsy. *Biosens Bioelectron* **168**, 112520 (2020).

10. H. L. Wang *et al.*, Expression of prostate-specific membrane antigen in lung cancer cells and tumor neovasculature endothelial cells and its clinical significance. *PLoS One* **10**, e0125924 (2015).

11. A. G. Wernicke *et al.*, Prostate-specific membrane antigen expression in tumor-associated vasculature of breast cancers. *APMIS* **122**, 482-489 (2014).

12. S. S. Chang *et al.*, Five different anti-prostate-specific membrane antigen (PSMA) antibodies confirm PSMA expression in tumor-associated neovasculature. *Cancer Res* **59**, 7 (1999).

13. Y. Yu *et al.*, Engineering of exosome-triggered enzyme-powered DNA motors for highly sensitive fluorescence detection of tumor-derived exosomes. *Biosens Bioelectron* **167**, 112482 (2020).

14. J. Chen *et al.*, Organization of protein tyrosine kinase-7 on cell membranes characterized by aptamer probe-based STORM imaging. *Anal Chem* **93**, 936-945 (2021).

15. D. Fan *et al.*, A polydopamine nanosphere based highly sensitive and selective aptamer cytosensor with enzyme amplification. *Chem Commun (Camb)* **52**, 406-409 (2016).

16. Y. Fan, L. Li, M. Lu, H. Si, B. Tang, In situ fluorescent profiling of living cell membrane proteins at a single-molecule level. *Chem Commun (Camb)* **55**, 4043-4046 (2019).

17. J. Jiang, Y. Yu, H. Zhang, C. Cai, Electrochemical aptasensor for exosomal proteins profiling based on DNA nanotetrahedron coupled with enzymatic signal amplification. *Anal Chim Acta* **1130**, 1-9 (2020).

18. C. Liu *et al.*, Low-cost thermophoretic profiling of extracellular-vesicle surface proteins for the early detection and classification of cancers. *Nat Biomed Eng* **3**, 183-193 (2019).

19. M. Piliarik, V. Sandoghdar, Direct optical sensing of single unlabelled proteins and super-resolution imaging of their binding sites. *Nat Commun* **5**, 4495 (2014).
